# Morphological and anatomical insights into *de novo* shoot organogenesis of *in vitro* ‘Delite’ rabbiteye blueberries

**DOI:** 10.1016/j.heliyon.2020.e05468

**Published:** 2020-11-13

**Authors:** Carolina Schuchovski, Bruno Francisco Sant'Anna-Santos, Raquel Cristina Marra, Luiz Antonio Biasi

**Affiliations:** a Pós-graduação em Produção Vegetal, Universidade Federal do Paraná, Rua dos Funcionários, 1540, 80035-050, Curitiba, PR, Brazil; b Laboratório de Anatomia e Biomecânica Vegetal, Departamento de Botânica, Setor de Ciências Biológicas, Universidade Federal do Paraná, Avenida Coronel Francisco H. dos Santos, 100, Centro Politécnico, Jardim das Américas, C.P. 19031, 81531-980, Curitiba, PR, Brazil; c Departamento de Botânica, Setor de Ciências Biológicas, Universidade Federal do Paraná, Avenida Coronel Francisco H. dos Santos, 100, Centro Politécnico, Jardim das Américas, C.P. 19031, 81531-980, Curitiba, PR, Brazil; d Departamento de Fitotecnia e Fitossanidade, Universidade Federal do Paraná, Rua dos Funcionários, 1540, 80035-050, Curitiba, PR, Brazil

**Keywords:** Ericaceae, Scanning electron microscopy (SEM), Light microscopy, Organogenesis, In vitro regeneration, *Vaccinium virgatum*, Horticulture, Plant growth, Plant physiology, Botany, Biotechnology

## Abstract

Blueberries are valued for their taste and their high nutritional benefits, including their antioxidant and anti-inflammatory properties. *In vitro* culturing is an alternative method for clonal propagation, and has been used in many biotechnological studies. Most blueberry research is concentrated on highbush and lowbush taxa (*Vaccinium corymbosum* and *Vaccinium angustifolium* respectively), with only limited investigations of rabbiteye cultivars (*Vaccinium virgatum*) that are more suitable for subtropical climates and regions with warmer winters as a result of climate change. There is therefore a need to determine *in vitro* protocols for that species and group of cultivars. We examined here adventitious shoot regeneration in the ‘Delite’ rabbiteye blueberry cultivar. Leaf explants were cultured *in vitro* in Woody Plant Medium (WPM), and the effects of different thidiazuron (TDZ) concentrations, the orientation of the leaf (adaxial or abaxial surface in contact with the medium), and two portions of the leaf segment (basal or apical) were examined. *De novo* shoot development was studied using light and scanning electron microscopy. All concentrations of TDZ used showed similar survival and regeneration rates; 0.5 μM TDZ showed high efficiency in regenerating adventitious shoots (100%, with 57 adventitious shoots/explant), as did the adaxial surface in contact with the medium using either the apical or the basal portion of the leaf (97% shoot regeneration, 47.5 adventitious shoots/explant). Anatomical analyses showed direct and indirect organogenesis. The shoots developed leaf primordia with stomata, trichomes, and well-developed vascular tissues, with further elongation and rooting of the plants. We therefore describe here a high-efficiency regeneration method through *de novo* shoot organogenesis using TDZ in foliar explants of rabbiteye blueberry, with direct and indirect organogenesis.

## 1 Introduction

Blueberry is a perennial fruit crop of the Ericaceae family and genus *Vaccinium*. The fruits offer high nutraceutical benefits, and show antioxidant and anti-inflammatory properties [1]. Blueberries are rich in polyphenol compounds that can induce neurogenesis in adults [2] with anti-inflammatory activity [3]. Additionally, blueberries have high concentrations of anthocyanins, with beneficial effects against chronic diseases such as cancer, diabetes, neurodegenerative diseases, and cardiovascular disorders [4], and have high concentrations of vitamin C [5].

The fact that blueberries have several bioactive compounds related to health benefits, in addition to their good taste makes them attractive to consumers – and production has been steadily increasing, with the commercialization of fresh fruits as well as juices, and frozen and dried processed products [1].

Multiple species are involved in the commercial production of blueberries, with the vast majority composed of *Vaccinium corymbosum* L. (tetraploid highbush blueberry) and its hybrids and *Vaccinium angustifolium* Ait. (tetraploid lowbush blueberry), with lesser quantities of *Vaccinium virgatum* Ait. (hexaploid rabbiteye blueberry) [6]. Increased demand has led to increases in blueberry production in different regions beyond its native origin – demanding new cultivars adapted to different environments. In warmer regions, rabbiteye blueberries have been shown to be a noteworthy alternative, with lower demands for cold and chilling hours to grow and produce.

Blueberry crops are mainly propagated vegetatively through cuttings, which can lead to pathogenic infections. Therefore, for best blueberry production, vegetative propagation should employ methods that assure phytosanitary standards. *In vitro* culture therefore represents an important method for blueberry clonal propagation, as it can potentially produce large numbers of plants and propagate newly released cultivars [7]. There has been a good deal of previous research on *in vitro* blueberry culturing, although much of it has been related to cultivars adapted to temperate climates. Numerous studies have focused on highbush and lowbush cultivars [8, 9, 10, 11, 12, 13, 14, 15, 16, 17], but only a few studies have focused on rabbiteye *in vitro* regeneration techniques [10].

The protocols already developed are specific to each genotype, and depend on suitable concentrations of growth regulators in the culture medium [8], indicating the importance of research into specific protocols for different genotypes. Therefore, specific techniques need to be developed for rabbiteye blueberry cultivars that are better adapted to warmer winter regions, with efficient *in vitro* regeneration protocols that could be used for mass propagation as well as for the development of other studies in biotechnology.

Different growth regulators used in culture media will elicit distinct morphogenic responses [8,18], and adventitious bud regeneration protocols for blueberries have employed cytokinins and auxins, such as IAA (indole-3-acetic acid), 2iP [2-isopentenyladenine; 6-(γ-γ-dimethylallylamino)-purine], TDZ (thidiazuron), NAA (α-naphthaleneacetic acid), zeatin [as reviewed in 18], and IBA (indole-3-butyric acid) [10].

Thidiazuron (TDZ) has been used in many *in vitro* culture protocols, and elicits effects similar to auxins and cytokinins [16]. It has been tested in the *in vitro* regeneration of some *Vaccinium* species [11,13,15,16,19, 20, 21, 22], as well as other genera, such as *Billbergia* [23], *Melastoma* [24], *Brassica* [25], *Cucumis* [26], *Populus* [27], *Arachis* [28], *Ficus* [29, 30, 31, 32, 33], *Morus* [34], *Chenopodium* [35] and *Lotus* [36].

TDZ has been widely employed in many *in vitro* techniques, such as micropropagation, and has been found to induce axillary proliferation at low concentrations. It can also be used at high concentrations (greater than 1 μM) for callus formation, organogenesis, and somatic embryogenesis. The high activity of TDZ can be explained by its lower susceptibility to enzymatic degradation as compared to natural cytokinins, and it can be useful with genotypes that are otherwise difficult to propagate, including woody species. Its use in high concentrations can lead to undesirable effects, however, such as reduced shoot elongation, hyperhydricity, and shoot fasciation. It is of significant importance to determine the optimal TDZ concentration (or combinations of TDZ with other growth regulators) required for efficient *in vitro* regeneration process [37].

We therefore sought to develop an efficient *in vitro* regeneration technique for ‘Delite’ rabbiteye blueberry through shoot organogenesis from leaf explants, to study the developmental process of the *de novo* formed shoots, and to address a number of questions: what TDZ medium concentration is most suitable for inducing adventitious shoot formation from leaf explants? Will leaf explant orientation and portions affect the results? Is organogenesis direct or indirect? Are *de novo* shoots well-formed?

## 2 Material and methods

### 2.1 Plant material

Leaf explants of the ‘Delite’ rabbiteye blueberry cultivar were collected from *in vitro* plants growing on WPM [38] supplemented with Murashige and Skoog (MS) organic compounds [39], 2.5 μM zeatin, and 30 g L^−1^ sucrose. All media were jellified with 7 g L^−1^ agar (Vetec, Rio de Janeiro/Brazil) after the pH was adjusted to 5.2. The media were then autoclaved at 120 °C and 1.0 atm for 20 min; the zeatin was sterilized through 0.22 μm filters and added to the cooled media. Cultures were maintained at 25 ± 2 °C under cool daylight at 40 μmol m^−2^ s^−1^ with a 16-h photoperiod.

### 2.2 Experiment with different TDZ concentrations in WPM culture medium

This organogenesis experiment was conducted using a completely randomized design, with six treatments representing different TDZ concentrations (0, 0.5, 1.0, 1.5, 2.0 and 2.5 μM). The medium was prepared using WPM culture medium supplemented with MS organic compounds, 30 g L^−1^ sucrose, and different TDZ concentrations. All media were jellified with 7 g L^−1^ agar (Vetec) after the pH was adjusted to 5.2. The media were then autoclaved at 120 °C and 1.0 atm and poured into sterilized Petri dishes (15 mL/dish). Leaf explants were collected from *in vitro* plants and placed in the Petri dishes with their adaxial surfaces in contact with the medium. Cultures were maintained in the culture room as described above. Each treatment used four replications (ten leaf explants in each replicate, placed in one Petri dish), for a total of 40 explants per treatment and a total of 240 leaf explants. Leaf explants were evaluated under a stereomicroscope ten weeks later, and scored according to their survival rate (%), shoot regeneration rate (%) (percentage of explants showing adventitious shoots), number of new shoots formed per explant (total number), and number of new shoots formed per explant considering their sizes (large, medium, or small). The shoot sizes were classified as: large, if longer than 1 mm and held leaves; medium, if shorter than 1 mm and held leaves; or small, if less than 1 mm long and did not bear any leaves. Contaminated cultures (0–30% of the explants) were not included in the statistical analyses. After the first evaluation, the explants were placed in fresh media (as previously described), with no TDZ, and supplemented with 2.5 μM zeatin.

### 2.3 Experiment with two explant orientations (adaxial or abaxial), and two leaf portions (basal or apical)

In this experiment, a two-factor (2 × 2) arrangement and a completely randomized design were used, with factor 1 being the different explant orientations (adaxial or abaxial surface in contact with the medium) and factor 2 being the leaf portion (basal or apical), in a total of four treatments. The medium used was WPM supplemented with MS organic compounds, 30 g.L^−1^ sucrose, and 1 μM TDZ. All media were jellified with 7 g L^−1^ agar (Vetec) after the pH was adjusted to 5.2. The medium was then autoclaved at 120 °C and 1.0 atm and placed in sterilized Petri dishes (15 mL/dish). Leaf explants were collected from *in vitro* plants and placed in the Petri dishes according to the arrangement of the different treatments: adaxial or abaxial surface in contact with the medium, and using the basal or apical portion of the leaf. The cultures were maintained in a culture room as previously described. Each treatment consisted of five replicates (ten leaf explants in each replication, placed in one Petri dish), for a total of 50 explants per treatment, and 200 total leaf explants. Ten weeks later the leaf explants were evaluated using a stereomicroscope, according to the same criteria mentioned earlier. There was no contamination in this experiment. After the first evaluation, explants were placed in fresh media as previously described, with no TDZ and supplemented with 2.5 μM zeatin.

### 2.4 Experimental design and statistical analysis

All of the experiments were conducted according to a completely randomized design. First, the means of the explants in each replication were calculated (evaluating all of the explants), and then the means of the four or five replicates in each treatment were calculated. Levene's test was performed to confirm the homogeneity of the variances among the treatments, and then analysis of variance (ANOVA) was performed to detect significant differences between treatments, and Tukey's multiple range test (p < 0.05) was used to identify the superior treatments. The results are presented as the mean ± standard error in the tables. In the experiment with different TDZ concentrations, linear regression analyses were performed with the variables confirmed to have statistical significance in the analysis of variance of the regression. Those variables were “number of new shoots formed per explant (total number)” and “number of new shoots formed per explant (small sized)”. All statistical analyses were performed using R software [40].

### 2.5 Morphoanatomical analyses

In these evaluations, the WPM culture medium was supplemented with MS organic compounds, 30 g.L^−1^ sucrose, and 1.0 μM TDZ. All media were jellified with 7 g L^−1^ agar (Vetec) after the pH was adjusted to 5.2. Subsequently, the media was autoclaved at 120 °C and 1.0 atm, and cultures were maintained at 25 ± 2 °C under cool daylight at 40 μmol m^−2^ s^−1^ with a 16-h photoperiod. Leaf explants were excised from *in vitro* plants and placed in Petri dishes containing 15 mL of culture medium. Each Petri dish contained ten leaf explants positioned with their adaxial surfaces in contact with the medium.

Ten leaf explants were collected at every stage weekly (from three- to seven-week-old culture), for a total of 70 explants. The developmental processes of *de novo* shoot organogenesis were observed using both light and scanning electron microscopy (SEM).

The aforementioned explants were observed, and photodocumentation was performed using a stereomicroscope. Samples were fixed in modified Karnovsky solution (2.5% glutaraldehyde and 10% paraformaldehyde in 0.1 M phosphate buffer, pH 7.2) [41].

For SEM, the fixed samples (as previously described) were dehydrated in an ethylic series. Critical point drying was obtained using a Bal-Tec CPD 030 Critical Point Dryer. Samples were fixed onto aluminum stubs and gold coated. The images were obtained using a JEOL JSM 6360-LV scanning electron microscope.

In the light microscopy analyses, after fixation, the samples were dehydrated in an ethylic series and embedded in methacrylate (Historesin, Leica Microsystems, Nussloch/Germany). The solidified blocks were sectioned (8 μm thick) in a rotary microtome (Olympus CUT 4055), the slides stained with 5% (w/v) toluidine blue [42], and subsequently photographed under a light microscope (Olympus BX51).

## 3 Results

*De novo* shoot organogenesis was achieved from blueberry leaf explants in WPM culture medium containing TDZ.

### 3.1 Experiment with different TDZ concentrations in WPM culture medium

**Figure 1:**
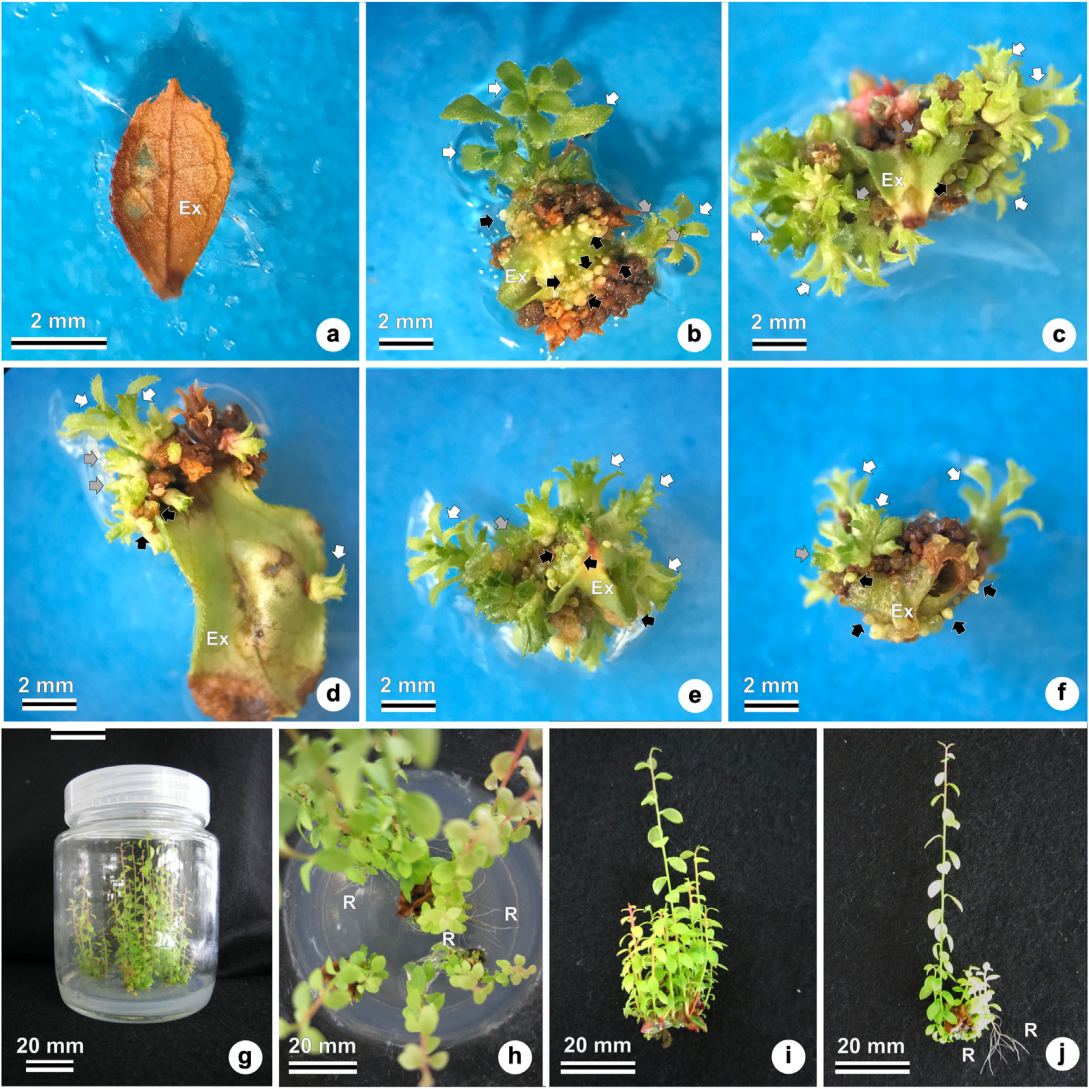
Effects of different TDZ concentrations on *in vitro* shoot organogenesis in ‘Delite’ rabbiteye blueberry using WPM culture medium. Different views of explants and shoots under a stereomicroscope (a–f) and digital camera (g–j). (a) TDZ 0 μM, showing an oxidized leaf explant. (b) TDZ 0.5 μM with many small (black arrow), medium (gray arrow), and large (white arrow) shoots. (c) TDZ 1.0 μM, with small (black arrow), medium (gray arrow), and large (white arrow) shoots. (d) TDZ 1.5 μM, with small (black arrow), medium (gray arrow), and large (white arrow) shoots. (e) TDZ 2.0 μM, with small (black arrow), medium (gray arrow), and large (white arrow) shoots. (f) TDZ 2.5 μM, with small (black arrow), medium (gray arrow), and large (white arrow) shoots. (g–j) Development of the shoots at 20 weeks after the first evaluation in fresh culture medium with 2.5 μM zeatin. Details of *in vitro* rooting in (h) and (j). Abbreviations: Ex, explant; R, roots; TDZ, thidiazuron; WPM, woody plant medium.

In this experiment, the explant survival rates were higher than 93%. The treatments with TDZ showed 100% explant survival, superior to the treatment with no TDZ. All the treatments containing TDZ showed 100% of the explants with shoot regeneration (Table 1), while the treatment without TDZ showed no regeneration (Figure 1). The analysis of variance is detailed in Table 2.

**Table 1:** Effects of different TDZ concentrations on *in vitro* shoot organogenesis in ‘Delite’ rabbiteye blueberry using WPM culture medium.

Treatment	Survival rate	Shoot regeneration rate	Number of new shoots formed/explant (large sized)	Number of new shoots formed/explant (medium sized)
	%	%	n.	n.
0 μM	93.3 ± 3.3 b	0.0 ± 0.0 b	NA	NA
0.5 μM	100.0 ± 0.0 a	100.0 ± 0.0 a	1.3 ± 0.6 a	5.4 ± 1.9 a
1.0 μM	100.0 ± 0.0 a	100.0 ± 0.0 a	3.0 ± 0.2 a	5.0 ± 0.7 a
1.5 μM	100.0 ± 0.0 a	100.0 ± 0.0 a	2.4 ± 1.1 a	5.4 ± 1.4 a
2.0 μM	100.0 ± 0.0 a	100.0 ± 0.0 a	2.4 ± 1.0 a	7.1 ± 1.5 a
2.5 μM	100.0 ± 0.0 a	100.0 ± 0.0 a	4.3 ± 1.5 a	6.9 ± 1.8 a
Mean	99.1	85.7	2.7	5.9
CV%	2.1	0.0	70.8	50.6

The results are presented as the mean ± standard error (SE). Means followed by different letters in the same column differ statistically at 5% of Tukey's multiple range tests. Abbreviations: CV, coefficient of variation; NA, not available; TDZ, thidiazuron; WPM, woody plant medium.

**Table 2:** Analysis of variance (ANOVA) of the experiments evaluating the effects of different thidiazuron (TDZ) concentrations on *in vitro* shoot organogenesis in ‘Delite’ rabbiteye blueberry. Abbreviations: DF, degrees of freedom; ms, mean squares; ns, non-significant; TDZ, thidiazuron.

	DF	Survival rate	Shoot regeneration rate		Number of new shoots formed/explant (total)	Number of new shoots formed/explant (large sized)	Number of new shoots formed/explant (medium sized)	Number of new shoots formed/explant (small sized)
		ms	ms	DF	ms	ms	ms	ms
Treatment	5	22.8	5142.9	4	572.5	4.8	3.239	697.7
Residuals	15	4.4	0.000	13	127.3	3.6	8.947	105.7
Total	20			17				
p-value		0.0060 ∗∗	<2.2e-16 ∗∗		0.0169 ∗	0.3190 ns	0.8313 ns	0.0040 ∗∗

∗ statistically different with 0.05 > p-value > 0.01.

∗∗ statistically different with p-value ≤ 0.01.

ns, non-significant, p-value ≥ 0.05.

**Figure 2:**
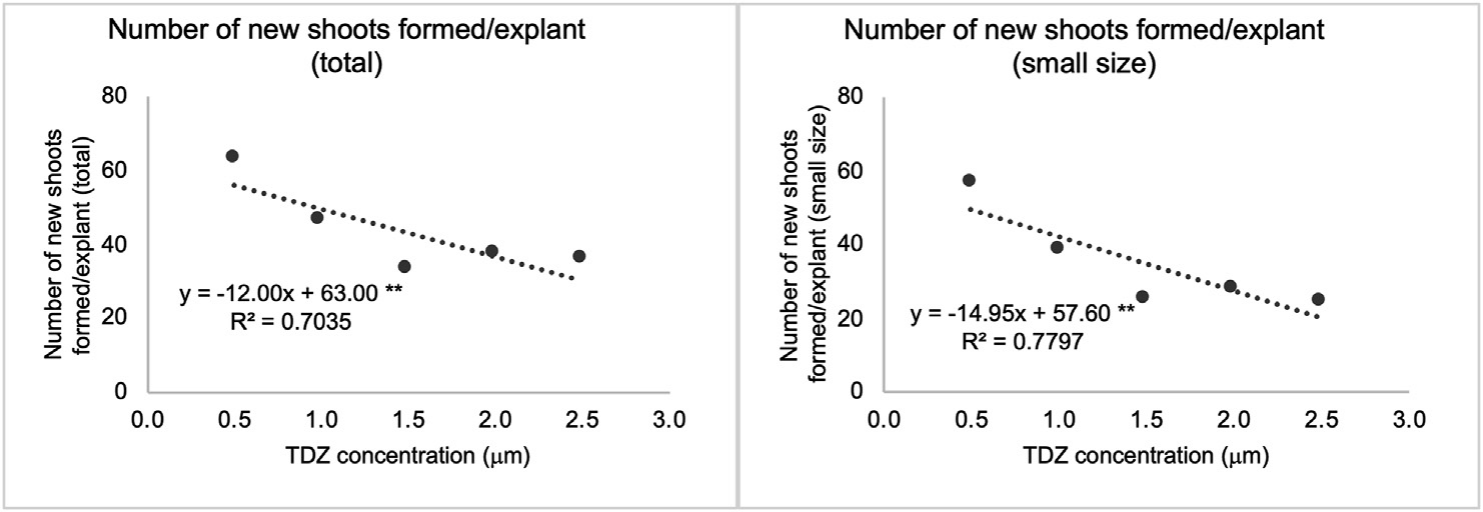
Effects of different TDZ concentrations on *in vitro* shoot organogenesis in ‘Delite’ rabbiteye blueberry using WPM culture medium. Simple linear regression graphics showing the dependent variables number of new shoots formed/explant (total) on the left, and the number of new shoots formed/explant (small size) on the right. ∗∗ statistically significant with p-value ≤0.01. Abbreviations: TDZ, thidiazuron; WPM, woody plant medium.

The numbers of new shoots formed per explant (total) were different between the treatments containing TDZ. A simple linear regression equation for that variable was statistically significant (Figure 2), and describes that for each 1 μM increase in TDZ concentration in the medium, there was a decrease of 12.0 shoots per explant. Estimated values varied from 57 to 33 new shoots formed per explant increasing concentrations of TDZ, from 0.5 to 2.5 μM.

If we separate the results concerning the number of new shoots formed into large, medium, and small sized classes (as previously described), a similar pattern can be recognized with small shoots. In this case, there was a decrease of 15.0 shoots at each increase of 1 μM of TDZ concentration (Figure 2), with estimated values ranging from 50.1 to 20.2 small shoots per explant with increasing TDZ concentrations (from 0.5 to 2.5 μM).

However, observing the newly formed large and medium sized shoots, there were no differences between the TDZ treatments (Table 2), with values ranging from 1.3 to 4.3 (large sized) and 5.0 to 7.1 (medium sized), with means of 2.7 and 5.9 new shoots per explant (large and medium sized respectively) (Table 1).

In Figure 1, *de novo* shoot formation through organogenesis can be observed in the six different treatments with TDZ (a to f) ten weeks after the initiation of culturing. Figure 1a shows an oxidized leaf explant and no shoot regeneration in the control treatment (without TDZ). Figures 1b–1f show the effects of different TDZ concentrations on leaf explants, regenerating small, medium, and large sized shoots.

Further shoot growth in fresh culture medium with 2.5 μM zeatin was observed 20 weeks after the first evaluations (Figure 1g–j), with subsequent *in vitro* rooting of the explants (Figure 1h, j).

### 3.2 Experiment with two explant orientations (adaxial or abaxial), and two leaf portions (basal or apical)

Analysis of variance showed that there were interactions between the factors of explant orientation and leaf portion only for the variables of number of new shoots formed per explant (total) and number of new shoots formed per explant (small sized); there were no interactions between the factors in terms of the variables survival rate, shoot regeneration rate, number of new shoots formed per explant (large sized), and number of new shoots formed per explant (medium sized) (Tables 3 and 4).

**Table 3:** Effects of two explant orientations (adaxial or abaxial side in contact with the medium) and two leaf portions (basal or apical) on *in vitro* shoot organogenesis in ‘Delite’ rabbiteye blueberry using WPM culture medium supplemented with TDZ.

Explant orientation	Leaf portion		
**Survival rate (%)**	Basal	Apical	Mean
Adaxial	100.0 ± 0.0	100.0 ± 0.0	100.0 a
Abaxial	82.0 ± 10.3	94.0 ± 4.5	88.0 b
Mean	91.0 A	97.0 A	94.0
**Shoot regeneration rate (%)**			
Adaxial	94.0 ± 4.5	100.0 ± 0.0	97.0 a
Abaxial	74.0 ± 12.0	94.0 ± 4.5	84.0 b
Mean	84.0 B	97.0 A	90.5
**Number of shoots formed/explant (total)**			
Adaxial	59.2 ± 15.4 aA	35.8 ± 3.8 aA	47.5
Abaxial	22.8 ± 3.1 bA	41.3 ± 9.6 aA	32.0
Mean	41.0	38.6	39.8
**Number of shoots formed/explant (large sized)**			
Adaxial	4.1 ± 0.7	4.5 ± 1.5	4.3 a
Abaxial	2.5 ± 0.5	1.1 ± 0.2	1.8 b
Mean	3.3 A	2.8 A	3.1
**Number of shoots formed/explant (medium sized)**			
Adaxial	9.0 ± 1.1	6.4 ± 1.1	7.7 a
Abaxial	4.0 ± 0.6	6.1 ± 1.8	5.1 a
Mean	6.5 A	6.3 A	6.4
**Number of shoots formed/explant (small sized)**			
Adaxial	46.1 ± 15.3 aA	24.9 ± 4.3 aA	35.5
Abaxial	16.2 ± 3.0 bA	39.5 ± 7.6 aA	27.9
Mean	31.2	32.2	31.7

The results are presented as the mean ± standard error (SE). Means followed by different lowercase letters in the same column and by different uppercase letters in the same horizontal line differ statistically at 5% of Tukey's multiple range tests. Abbreviations: TDZ, thidiazuron; WPM, woody plant medium.

**Table 4:** Analysis of variance (two-way ANOVA) of the experiments evaluating the effects of explant orientation (adaxial or abaxial side in contact with the medium) and the leaf portion (basal or apical) on *in vitro* shoot organogenesis in ‘Delite’ rabbiteye blueberry. Abbreviations: CV, coefficient of variation; DF, degrees of freedom; ms, mean squares.

	Source	DF	ms	p value
Survival rate (%)	Explant orientation	1	720.00	0.0289 ∗
	Leaf portion	1	180.00	0.2476 ns
	Interaction (explant orientation x leaf portion)	1	180.00	0.2476 ns
	Residuals	16	125.00	
CV: 11.9%	Total	19		
Shoot regeneration rate (%)	Explant orientation	1	845.00	0.0484 ∗
	Leaf portion	1	845.00	0.0484 ∗
	Interaction (explant orientation x leaf portion)	1	245.00	0.2667ns
	Residuals	16	185.00	
CV: 15.0%	Total	19		
Number of shoots formed/explant (total)	Explant orientation	1	1196.60	0.0844 ns
	Leaf portion	1	29.40	0.7766 ns
	Interaction (explant orientation x leaf portion)	1	2199.10	0.0239 ∗
	Residuals	16	353.40	
CV: 47.3%	Total	19		
Number of shoots formed/explant (large size)	Explant orientation	1	31.25	0.0053 ∗∗
	Leaf portion	1	1.36	0.5100 ns
	Interaction (explant orientation x leaf portion)	1	3.93	0.2696 ns
	Residuals	16	3.00	
CV: 56.5%	Total	19		
Number of shoots formed/explant (medium size)	Explant orientation	1	34.45	0.0312 ∗
	Leaf portion	1	0.26	0.8407 ns
	Interaction (explant orientation x leaf portion)	1	27.31	0.0516 ns
	Residuals	16	6.17	
CV: 39.9%	Total	19		
Number of shoots formed/explant (small size)	Explant orientation	1	289.18	0.3561 ns
	Leaf portion	1	5.63	0.8962 ns
	Interaction (explant orientation x leaf portion)	1	2478.21	0.0133 ∗
	Residuals	16	320.16	
CV: 56.5%	Total	19		

∗ significant different with 0.05 > p-value > 0.01.

∗∗ significant different with p-value ≤ 0.01.

ns, non-significant, p-value ≥ 0.05.

The basal or apical leaf portion treatments showed no differences in their survival rate, with 91.0 and 97.0% of explants surviving respectively (Table 3). A difference was observed, however, between the adaxial and abaxial sides of the explant in contact with the medium, with the adaxial orientation achieving 100% survival, and the abaxial orientation only 88%.

The highest shoot regeneration rate occurred when the explant orientation was adaxial (97.0%), and the leaf portion apical (97.0%).

When the basal portion of the leaf was cultured, the variables of number of shoots formed per explant (total) and number of shoots formed per explant (small sized), using an adaxial placement, were found to be superior (59.2 total shoots, and 46.1 small shoots) to an abaxial orientation (22.8 total shoots, and 16.2 small shoots). When the apical portion was used, no differences were observed between the adaxial or abaxial orientations in terms of the variables of: number of shoots formed per explant (total number) and number of shoots formed per explant (small sized) (Table 3). In the treatments using the adaxial side in contact with the medium, there was no difference between apical and basal portions in terms of the total number of shoots and the number of small shoots per explant.

The adaxial positioning of the leaf on the medium resulted in larger numbers of large shoots formed per explant (4.3 shoots) than the abaxial orientation (1.8 shoots), although no differences were observed between the basal and apical leaf portions.

No differences were observed between the numbers of new medium sized shoots formed per explant, with an overall mean of 6.4 (Table 3).

**Figure 3:**
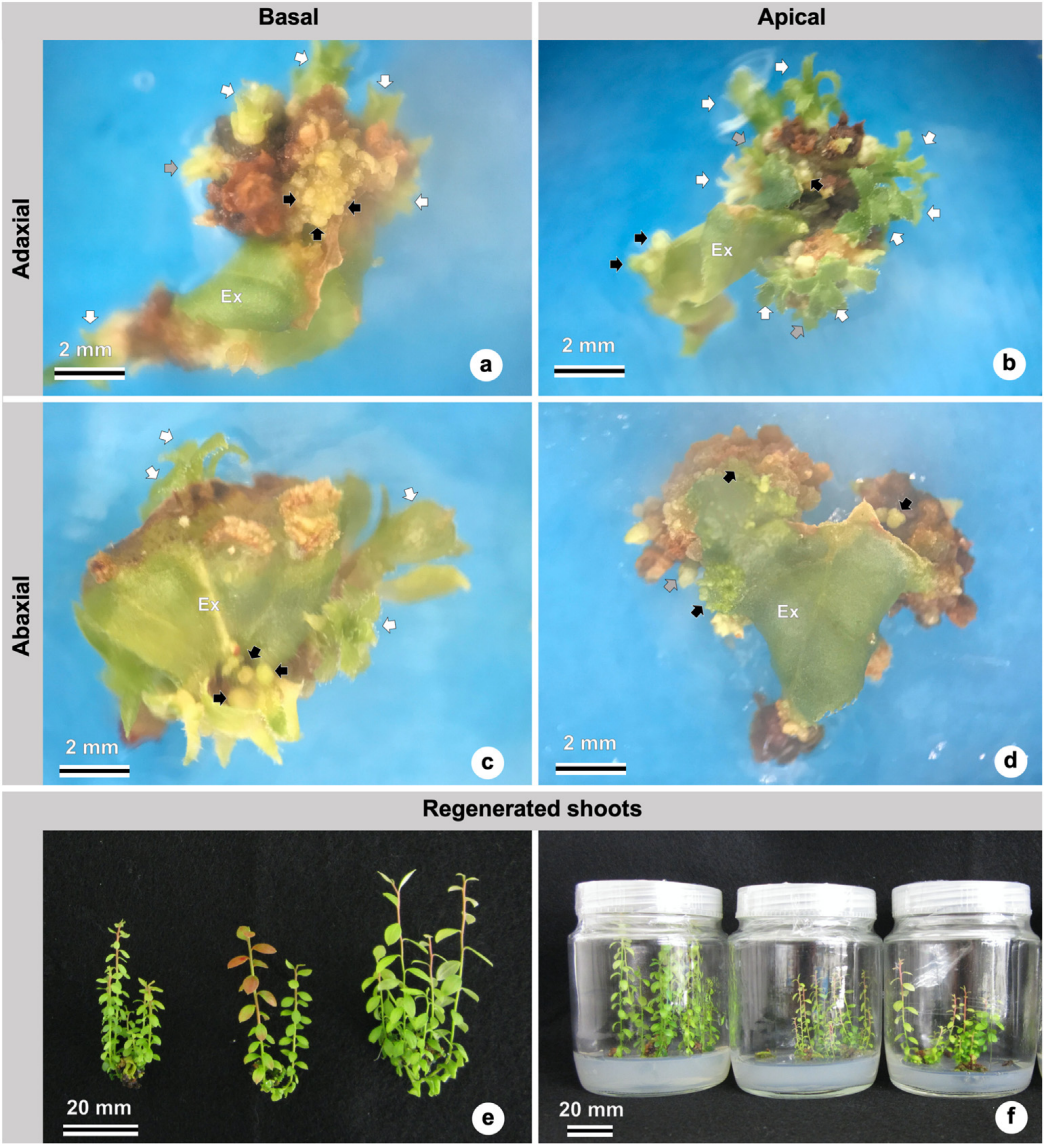
Effects of two explant orientations (adaxial or abaxial side in contact with the medium) and two leaf portions (basal or apical) on *in vitro* shoot organogenesis in ‘Delite’ rabbiteye blueberry using WPM culture medium supplemented with TDZ. Different views of explants and shoots under a stereomicroscope (a–d) and digital image capturing (e–f). (a) Adaxial x Basal, with many small (black arrow), medium (gray arrow), and large (white arrow) shoots. (b) Adaxial x Apical, with many small (black arrow), medium (gray arrow), and large (white arrow) shoots: (a, b) Those two adaxial treatments showed the best efficiency in regenerating shoots. (c) Abaxial x Basal with small (black arrow) and large (white arrow) shoots. (d) Abaxial x Apical, with small (black arrow) and medium (gray arrow) shoots. (e) Regenerated shoots after adventitious organogenesis. (f) *In vitro* regenerated shoots after adventitious organogenesis, subcultured into WPM culture medium supplemented with 2.5 μM zeatin, pictured ten weeks later. Abbreviations: TDZ, thidiazuron; WPM, woody plant medium.

In Figure 3, regenerating shoots can be seen forming over the leaf explant in the four treatments (Figure 3a–d), and, ten weeks later, the shoots can be seen growing in the WPM medium supplemented with 2.5 μM zeatin but without TDZ (Figure 3e, f).

### 3.3 Morphoanatomical analyses

**Figure 4:**
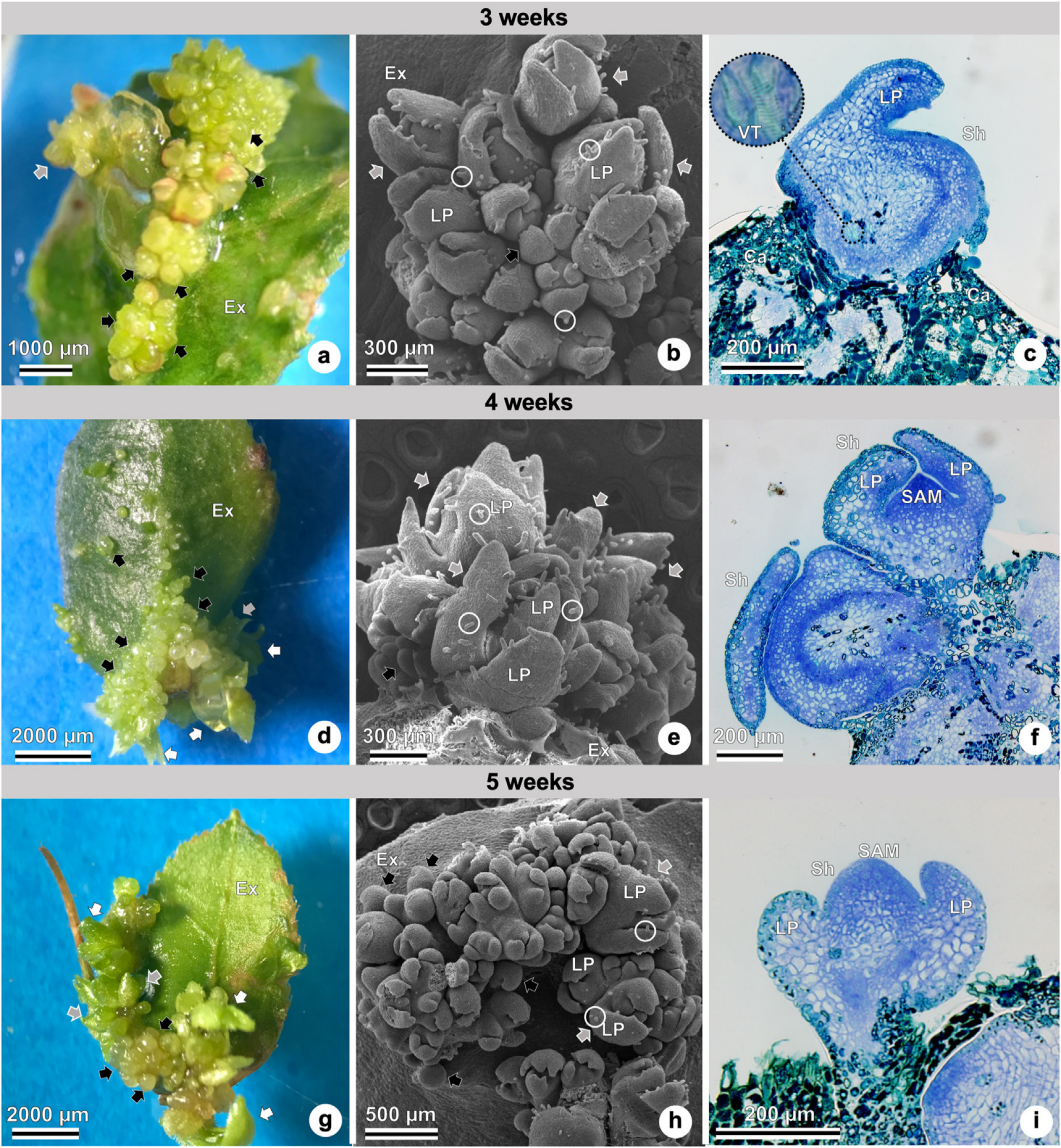
*In vitro* shoot organogenesis in ‘Delite’ rabbiteye blueberry using WPM culture medium supplemented with TDZ at different times (three- to five-week culture). Different views of explants and shoots under a stereomicroscope (a, d, and g), scanning electron microscope–SEM (b, e, and h), and light microscope (c, f, and i). (a) Three weeks of culture, leaf explant with small (black arrow), medium (gray arrow), and large (white arrow) shoots. (b) Three weeks of culture, leaf explant with small (black arrow), and medium (gray arrow) shoots, showing leaf primordia with trichomes (white circle). (c) Three weeks of culture, leaf explant with shoot, leaf primordium, vascular tissue (in detail), and callus formation. (d) Four weeks of culture, leaf explant with small (black arrow), medium (gray arrow), and large (white arrow) shoots. (e) Four weeks of culture, leaf explant with small (black arrow), and medium (gray arrow) shoots, showing leaf primordia with trichomes (white circle). (f) Four weeks of culture, shoots with leaf primordia, and shoot apical meristem. (g) Five weeks of culture, leaf explant with small (black arrow), medium (gray arrow), and large (white arrow) shoots. (h) Five weeks of culture, leaf explant with small (black arrow), and medium (gray arrow) shoots, showing leaf primordia with trichomes (white circle). (i) Five weeks of culture, shoots with shoot apical meristem and leaf primordia. Abbreviations: Ca, callus; Ex, explant; LP, leaf primordium; SAM, shoot apical meristem; Sh, shoot; TDZ, thidiazuron; VT, vascular tissue; WPM, woody plant medium.

**Figure 5:**
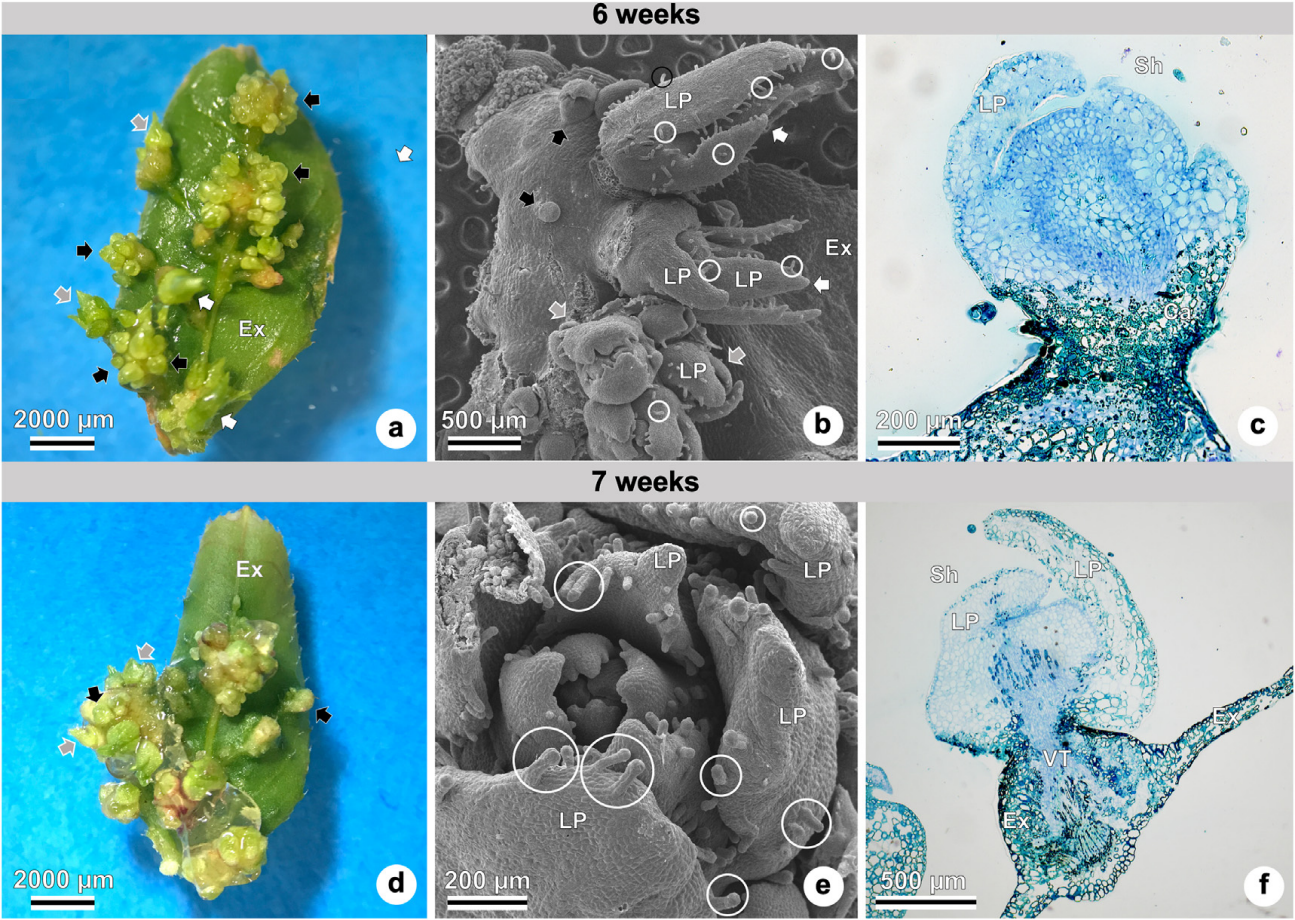
*In vitro* shoot organogenesis in ‘Delite’ rabbiteye blueberry using WPM culture medium supplemented with TDZ at different times (six and seven-week-old cultures). Different views of explants and shoots under a stereomicroscope (a and d), scanning electron microscope–SEM (b and e), and light microscope (c and f). (a, b, and c) Six-week-old culture. (d, e, and f) Seven-week-old culture. (a) Adaxial surface of the explant with small (black arrow), medium (gray arrow), and large (white arrow) shoots. (b) Explant with small (black arrow), medium (gray arrow), and large (white arrow) shoots, and leaf primordia with trichomes (white circle). (c) Shoot with leaf primordium, in indirect organogenesis: shoot formation originated from callus. (d) Adaxial surface of the explant with small (black arrow) and medium (gray arrow) shoots. (e) Shoot with several leaf primordia, showing trichomes (white circle). (f) Transversal view of the explant, with longitudinal view of the shoot formation through direct organogenesis, connecting to the explant vascular tissue. Abbreviations: Ca, callus; Ex, explant; LP, leaf primordium; Sh, shoot; TDZ, thidiazuron; VT, vascular tissue; WPM, woody plant medium.

**Figure 6:**
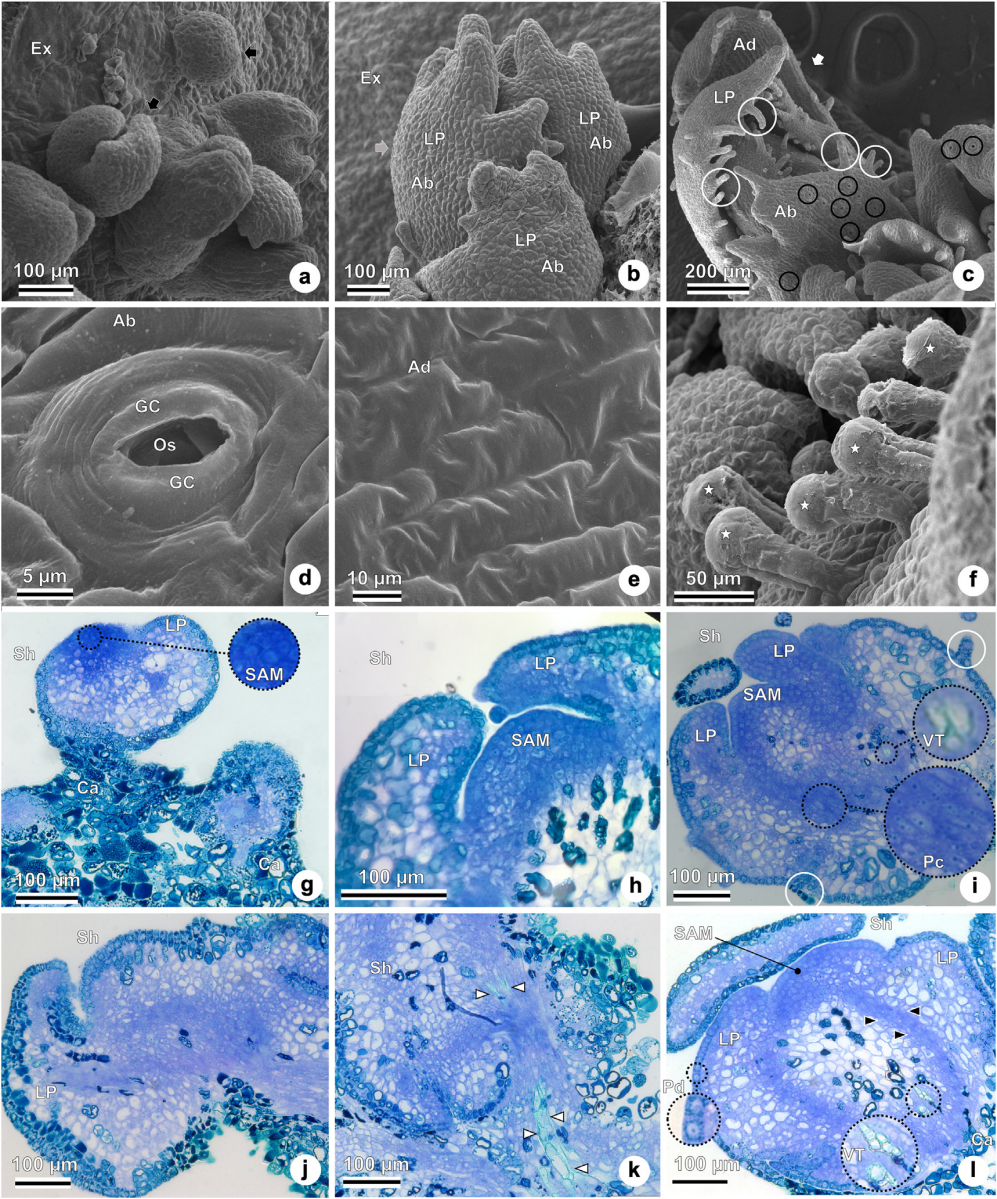
Details of *in vitro* shoot organogenesis in ‘Delite’ rabbiteye blueberry using WPM culture medium supplemented with TDZ. Different views of shoots and details under scanning electron microscopy–SEM (a–f) and light microscopy (g–l). (a) Newly formed adventitious small shoots (black arrow) on the surface of the three-week-old leaf explant culture. (b) Adventitious shoot (gray arrow) on a four-week-old leaf explant culture, showing leaf primordia (abaxial side visible). (c) Adventitious shoot (white arrow) on a four-week-old leaf explant culture, showing foliar primordia with stomata (black circle) and trichomes (white circle). Abaxial and adaxial surfaces of the leaf primordia visible. (d) Stomata on the abaxial surface of the leaf primordium: ostiole surrounded by guard cells. (e) Adaxial surface of the leaf primordium showing no stomata. (f) Trichomes on the leaf primordium (stars). (g) Adventitious shoots at three weeks of culture: shoot apical meristem (in detail), leaf primordium, and callus. (h) Adventitious shoot with leaf primordium and shoot apical meristem at four weeks of culture. (i) Detail of the adventitious shoot, showing shoot apical meristem, leaf primordium, procambium (detail), vascular tissue (detail), and trichomes (white circles) at four weeks of culture. (j) Leaf primordium formation at four weeks of culture. (k) Adventitious shoot showing the formation of vascular tissue (white ar) at four weeks of culture (l) Adventitious shoot with shoot apical meristem, leaf primordia with protoderm (detail), procambium (black ar), vascular tissue (detail) at 4 weeks of culture, and callus tissue. Abbreviations: Ab, abaxial; Ad, adaxial; Ca, callus; Ex, explant; GC, guard cell; LP, leaf primordium; Os, ostiole; Pc, procambium; Pd, protoderm; SAM, shoot apical meristem; Sh, shoot; TDZ, thidiazuron; Tr, trichome; VT, vascular tissue; WPM, woody plant medium.

*De novo* shoot organogenesis can be observed in Figures 4, 5, and 6 after three to seven weeks of culture. Three-week-old cultures show leaf explants with shoots (Figure 4a–c), followed by four-week-old cultures (Figure 4d–f), five-week cultures (Figure 4g–i), six-week cultures (Figure 5a–c), and seven-week cultures (Figure 5d–f).

The adaxial surface of the leaf explant can be seen with many adventitious small, medium, and large shoots in Figures 4a, 4d, 4g, 5a, 5d. The *de novo* shoots formed appear green when observed under a stereomicroscope, a feature indicative of the presence of chloroplasts in the epidermal cells.

Shoot organogenesis can be observed on the leaf explants, with recognizable leaf primordia – many of them already bearing glandular trichomes (Figures 4b, 4e, 4h, 5b, 5e). Figure 5e presents a top view of a forming shoot with many leaf primordia. The oldest leaf primordia are located along the outermost region of the shoot, while the youngest leaf primordia formed are located along the inner region of the shoot.

The development of adventitious shoots with leaf primordia can be observed in Figures 4c, 4f, 4I, 5c, 5f. Figure 4 c shows an adventitious shoot with leaf primordium being formed, and those shoots already show vascular tissue. Figures 4f and 4i highlight the dome-shaped shoot apical meristem with meristematic characteristics. That region could be recognized in histological observations by its small isodiametric cells with dense cytoplasm and large nuclei (Figures 4f, 4i, 6g, 6h, 6i, 6l).

Indirect organogenesis is evidenced by shoot formation from callus cells (Figure 5c), with disorganized aspects and green staining by toluidine blue. In Figure 5f, on the other hand, direct organogenesis is confirmed by the observation of shoot formation directly from the explant, with no callus cells. Additionally, the connections between the vascular tissue of the leaf explant with the adventitious shoot indicate direct organogenesis (Figure 5f).

Details of SEM images show (Figures 6a–6f) of three-to seven-week-old leaf explant cultures, with newly formed adventitious shoots easily visible (Figures 6a, 6b). Figure 6b shows the abaxial surface of the leaf primordia. More advanced stages are shown of the adaxial and abaxial surfaces of the leaf primordia, with numerous stomata on the abaxial surface and well-formed trichomes (Figure 6c). Detailed views of the stomata formed on the abaxial surface of the leaf primordium (Figure 6d) show opened ostioles surrounded by guard cells. The absence of stomata on the adaxial surface of the leaf primordium indicated that blueberry leaves are hypostomatic (Figure 6e). Glandular trichomes on blueberry leaves with evident secretory heads can be seen in Figure 6f.

Adventitious shoots, with details such as the shoot apical meristem and leaf primordium formation can be seen after three weeks of culturing (Figure 6g), with recognizable callus. Figure 6h shows details of the adventitious shoot with leaf primordium, evidence of a shoot apical meristem at four weeks of culturing, and tissue arrangements.

Figure 6i shows an adventitious shoot with leaf primordia, shoot apical meristem, procambium, vascular tissue, and trichomes on the leaf primordia. Figure 6j shows a shoot with leaf primordia. Figure 6k sows an adventitious shoot with the formation of vascular tissue after four weeks of culture. Figure 6l shows an adventitious shoot with an apical meristem, procambium, vascular tissue, and leaf primordia with protoderm, after four weeks of culture.

## 4 Discussion

**Figure 7:**
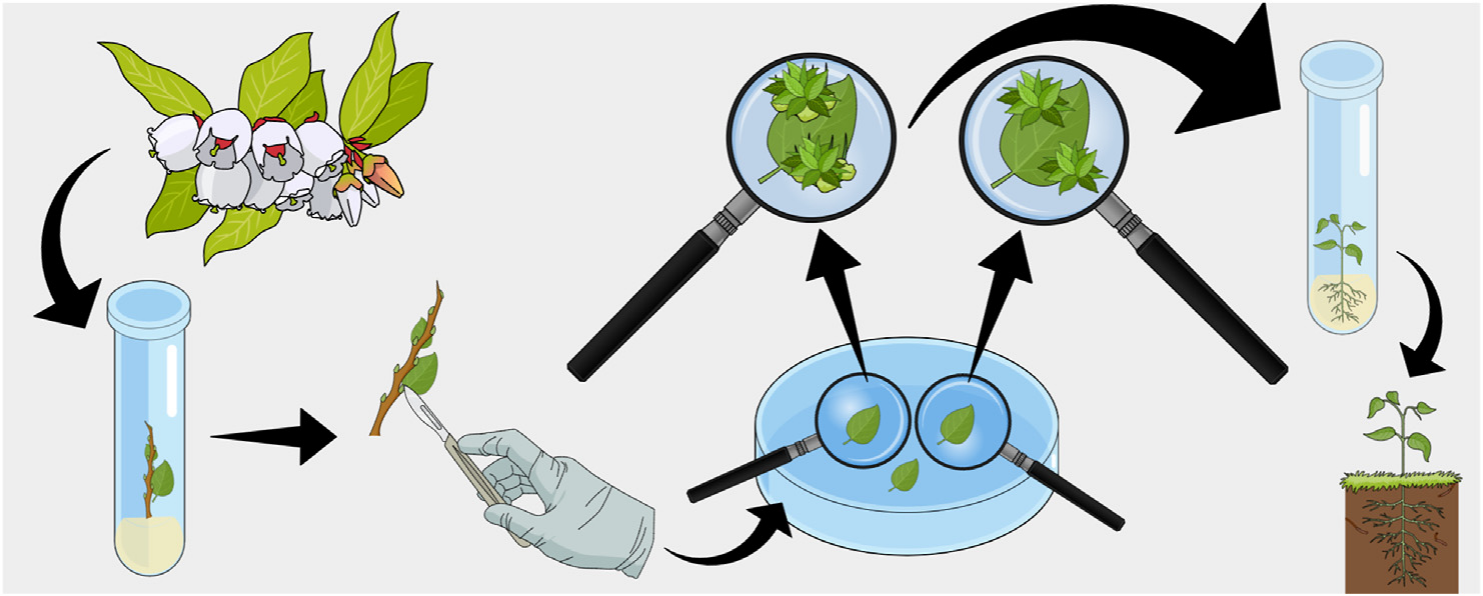
Diagrammatic representation of *de novo* shoot organogenesis in *in vitro* rabbiteye blueberry from leaf explants.

This study describes *de novo in vitro* shoot formation from leaf explants of ‘Delite’ rabbiteye blueberry, in the development of an important *in vitro* culture technique (Figure 7). We described the morphological and anatomical aspects of the developing shoots of blueberry based on light microscopy and SEM images. *De novo* shoot organogenesis is an example of a dedifferentiation process, where mature plant cells are capable of undergoing a reversible process from a mature and differentiated state to a meristematic stage [43].

Adjusting plant growth regulators in culture media is one of the most common approaches used in developing regeneration protocols such as somatic embryogenesis [44,45] and shoot proliferation [46,47]. TDZ is a potent cytokinin-like growth regulator that also shows auxin-like activity [16], and is a powerful plant growth regulator for establishing regeneration protocols.

We were able to regenerate shoots by incorporating TDZ into the culture medium, and found that the low concentration of 0.5 μM proved to be effective in adventitious shoot formation in ‘Delite’ blueberry. Other studies of *Vaccinium* reported that concentrations higher than 0.5 μM TDZ were more effective, with 0.5 mg L^−1^ (2.27 μM) TDZ inducing the highest number of shoots in the blueberry cultivar ‘Duke’ as compared to the lowest concentrations tested [0.1 mg L^−1^ (0.45 μM) and 0.2 mg L^−1^ (0.91 μM)] [8]. In another study with lowbush blueberry (*V. angustifolium*), the use of 2.3–4.5 μM TDZ allowed adventitious bud differentiation and shoot formation [13].

Some authors have examined organogenesis in blueberries using combinations of TDZ and other growth regulators (zeatin, zeatin riboside, and NAA), or even without TDZ, in studies of adventitious regeneration in different blueberry cultivars [15], and concluded that the optimum combinations of growth regulators were cultivar-dependent. In a study [10] with ‘Bluejay’ (highbush, *V. corymbosum*), ‘Pink Lemonade’ (rabbiteye derivative hybrid, *V. virgatum*), ‘Sunshine Blue’ (highbush, *V. corymbosum*), and ‘Top Hat’ (highbush x lowbush cross) cultivars, adventitious shoots were regenerated in culture media supplemented with different combinations of zeatin and IBA. A study with cranberry (*Vaccinium macrocarpon*) reported maximum regeneration rates in medium containing 10.0 μM TDZ with 1.0 μM NAA [19]. Another study with cranberry (*V. macrocarpon*) found that 10.0 μM TDZ with 5.0 μM 2iP was effective in initial adventitious regeneration [20].

The use of TDZ whenever possible as an alternative growth regulator to substitute the more commonly used zeatin will have the benefit of lowering the costs of *in vitro* blueberry culture [8].

We observed that *de novo* shoot cultures formed with TDZ in ‘Delite’ did not continue growing unless they were transferred to fresh medium supplemented with zeatin. It is known that TDZ can inhibit shoot elongation [19,37], so in order to assure shoot regeneration in lowbush blueberry (*V. angustifolium*), cultures initiated in TDZ must be transferred to a new medium containing zeatin (2.3–4.6 μM) to allow shoot elongation [13].

Leaf orientation, and the portion of it that is used, have been studied in *in vitro* organogenesis. A study with lowbush blueberry (*V. angustifolium*) found that basal leaves with their adaxial surface in contact with the medium proved to be most effective [12], and shoot apices were likewise found to form from the adaxial surfaces of leaf explants of ‘Aurora’ highbush *V. corymbosum* [11].

We found that both the apical and basal portions of the leaves generated high numbers of shoots per explant, but that the adaxial surface in contact with the medium resulted in higher survival and shoot regeneration rates, and great numbers of large shoots formed per explant. In evaluating the differences between adaxial and abaxial surfaces, considering the use of the basal leaf portion and the variable numbers of new shoots formed (total number and small sized shoots), higher yields were observed with the adaxial leaf surface in contact with the medium as compared to the abaxial surface. The observation that the adaxial surface in contact with the medium produced more shoots could be related to the fact that the abaxial side does not settle and completely enter in contact with the medium (as much as the adaxial treatments), due to its concave curvature.

Most of the shoots formed in our work appeared on the adaxial surface of the leaf explant, as was also reported with ‘Aurora’ (*V. corymbosum*) [11] and cranberry (*V. macrocarpon*) adventitious regeneration [20].

Both direct and indirect organogenesis were observed in this study, as shoots could originate directly from the leaf tissue of the explant with no apparent or histological evidence of callus formation, giving rise to direct organogenesis (Figure 5f), with connections between the vascular tissue of the new shoot with that of the leaf explant with no callus tissue being observed. The shoot formed in Figure 5c, on the other hand, originated from callus tissue, in a process of indirect organogenesis.

Callus proliferation is a process of unstructured cell division and enlargement, usually initiated from parenchymatous cells, and the cell walls typically contain secondary metabolites such as suberin, lignin, or phenolics [43]. In work with ‘Troyer’ citrange shoot regeneration, callus cells were found to evidence some lignification in cases of either direct or indirect organogenesis [48].

Callus tissue could be recognized in our work by its disorganized aspect, with a certain disaggregation and green staining with toluidine blue, generally indicating phenolic compounds in the cells [49]. Feder and O'Brien (1968) reported that toluidine blue will stain polyphenol containing cells a green color [50]; two studies with *Spondias dulcis* likewise reported that accumulations of phenolic compounds in the cells were stained green by toluidine blue [51,52], and the same staining was observed in a study with *Brassica oleracea* [53].

Various studies of *Vaccinium* adventitious shoot regeneration have reported either direct and/or indirect organogenesis; shoot apices of ‘Aurora’ highbush (*V. corymbosum*), were observed to form directly from parenchyma cells on the surface of leaf explants [11], and histological studies showed organogenesis without callus formation that initiated in sub-epidermal cells in highbush blueberry (*V. corymbosum*) [54].

Indirect organogenesis has been observed in ‘Bluejay’ (highbush, *V. corymbosum*), ‘Pink Lemonade’ (rabbiteye derivative hybrid, *V. virgatum*), ‘Sunshine Blue’ (highbush, *V. corymbosum*), and ‘Top Hat’ (highbush x lowbush cross) cultivars, with callus being induced from the explants, followed by adventitious shoot regeneration [10]. Callus formation was also observed in a somatic embryogenesis study with blueberry cultivars (*V. corymbosum* x *V. angustifolium*), with embryo development without the callus phase in a culture medium containing TDZ [16].

Similar to what we observed with the ‘Delite’ cultivar, direct and indirect organogenesis was obtained from leaf explants using ‘Duke’ highbush blueberry [8]. Additionally, in a study with lowbush blueberry (*V. angustifolium*), adventitious bud and shoot formation was observed with or without an intermediary callus phase [13].

Among other morphoanatomical characteristics, we identified dome-shaped shoot apical meristems under light microscopy with diameters varying from 120 to 200 μm, similar to the description of the shapes and sizes of shoot apical meristems in highbush field-grown blueberry (approximately 120 μm) [55].

Additional meristematic characteristics observed here, such as protoplasts strongly stained by toluidine blue, are in accordance with the literature [50].

We observed that leaf primordia were initiated along the flanks of the shoot meristem, which is in agreement with other studies [43]. Blueberries have simple leaves that are arranged alternately along the stem [55,56]. SEM images provided here show some details of leaf primordia formation (Figure 5e).

A study of the leaf anatomy of field-grown *V. corymbosum* showed their leaves to be bifacial, with all the stomata on the abaxial side of the leaf (hypoestomatic) [57]. An anatomical study of highbush blueberry leaves (*V. corymbosum*, cv. 'Bluetta') reported that stomata were present only on the abaxial surfaces of field-grown leaves, but they were observed on both surfaces of *in vitro* leaves [58] – differing from our findings with rabbiteye ‘Delite’ *in vitro* organogenesis, where only the abaxial surfaces of the leaf primordia held stomata. Therefore, the leaves of the shoots formed in our work demonstrated characteristics similar to those of field-grown plants, with their stomata restricted only to the abaxial surface – a common feature in blueberry plants.

We did not observe any signs of tissue hyperhidricity, which represents an essential achievement of our tissue culture work. Hyperhidricity is always a concern in *in vitro* culture, as it can limit subculturing and acclimatization survival, and represents a serious problem for tissue culturing, including for propagation, germplasm conservation, and plant breeding [59]. Hyperhidricity represents an alteration of the plant's normal morphophysiological state, with high water accumulation in the tissues and the formation of abnormal organs with water-soaked appearances [60]. Hyperhydric plants show discontinuous epidermal development, irregular stomatal formation, decreased stomatal density, intercellular spaces in the mesophyll, and reduced chlorophyll contents [59]. Blueberry cultivars (*Vaccinium* spp.) cultivated *in vitro* and showing hyperhydricity have a glassy aspect with translucent stems and leaves that are shortened and brittle, with deformed glandular trichomes, rough and crinkly epidermal, damaged stomata guard cells, enlarged mesophyll, disintegrated cell contours, deformed nuclei, and more intercellular spaces [59].

Morphological and anatomical analyses of the *de novo* shoots produced here showed them to be well-developed and with indicators of high viability, such as the green color of their shoots, well-developed and un-deformed stomata, glandular trichomes, shoot apical meristems, and leaf primordia, and cells with regular contours and well-delimited intercellular spaces. During the processes of sample preparation for microscopic examination the cells did not hardly dehydrate (the opposite of what would be expected with hyperhydric tissues), and the shoots were not glassy or translucent. Additionally, when the shoots were transferred to fresh medium with zeatin and without TDZ, they were able to survive, elongate, and form roots.

It is important to note that the morphogenic pathway observed here was of *de novo* shoot organogenesis from somatic cells in the leaf explant, developing a unipolar structure, and somatic embryogenesis (bipolar structure) was not observed. According to a study in *Passiflora* [61], changes of the auxin/cytokinin ratios can trigger those different developmental pathways; the authors observed both routes, but concluded that *de novo* shoot organogenesis generally occurred with exposure to a high cytokinin-to-low auxin ratio, or with cytokinin alone. Our study used only the cytokinin TDZ in the culture medium (although that growth regulator possibly have auxinic activity).

Adventitious shoot development stages are described here, showing that ‘Delite’ blueberry can demonstrate either direct or indirect organogenesis, with well-developed shoot apical meristems and leaf primordia. The leaf primordia of *de novo* shoots showed laminar shapes and a green color, with well-developed stomata and trichomes; adventitious shoots, and epidermal, parenchymatic, and vascular tissues were observed, with eventual shoot elongation, root formation, and the development of the whole plants.

## 5 Conclusion

The results presented here contribute to a better understanding of the *in vitro* organogenesis process in 'Delite' rabbiteye blueberry, and indicated a TDZ concentration of 0.5 μM in the WPM medium, using either the apical or the basal portions of the leaf and its adaxial surface orientation in contact with the medium. Both direct and indirect organogenesis were observed in that cultivar. The adventitious shoots showed the development of normal leaf tissues, and they grew and developed into rooted plants. Due to the high rate of regenerating explants and high numbers of shoots formed per explant, the techniques we describe here could be used for *in vitro* clonal propagation once genetic stability is confirmed. Additionally, it is expected that this research can help elucidate *in vitro* organogenesis regeneration process of ‘Delite’ rabbiteye blueberry plants, and contribute to further developing the biotechnology of blueberry cultivation.

## Declarations

### Author contribution statement

Carolina Schuchovski: Conceived and designed the experiments; Performed the experiments; Analyzed and interpreted the data; Contributed reagents, materials, analysis tools or data; Wrote the paper.

Bruno Francisco Sant'Anna-Santos: Conceived and designed the experiments; Analyzed and interpreted the data; Contributed reagents, materials, analysis tools or data; Wrote the paper.

Raquel Cristina Marra: Performed the experiments; Contributed reagents, materials, analysis tools or data.

Luiz Antonio Biasi: Conceived and designed the experiments; Analyzed and interpreted the data; Contributed reagents, materials, analysis tools or data; Wrote the paper.

### Funding statement

This research did not receive any specific grant from funding agencies in the public, commercial, or not-for-profit sectors.

### Data availability statement

Data included in article.

### Declaration of interests statement

The authors declare no conflict of interest.

### Additional information

No additional information is available for this paper.
